# Thermoelectric Nanocomposite Foams Using Non-Conducting Polymers with Hybrid 1D and 2D Nanofillers

**DOI:** 10.3390/ma11091757

**Published:** 2018-09-18

**Authors:** Mohammadmehdi Aghelinejad, Siu Ning Leung

**Affiliations:** Department of Mechanical Engineering, Lassonde School of Engineering, York University, Toronto, ON M3J 1P3, Canada; mehdi14@yorku.ca

**Keywords:** thermoelectric, polymer foams, carbon nanotubes, graphene, electrical conductivity, nanocomposite

## Abstract

A facile processing strategy to fabricate thermoelectric (TE) polymer nanocomposite foams with non-conducting polymers is reported in this study. Multilayered networks of graphene nanoplatelets (GnPs) and multi-walled carbon nanotubes (MWCNTs) are deposited on macroporous polyvinylidene fluoride (PVDF) foam templates using a layer-by-layer (LBL) assembly technique. The open cellular structures of foam templates provide a platform to form segregated 3D networks consisting of one-dimensional (1D) and/or two-dimensional (2D) carbon nanoparticles. Hybrid nanostructures of GnP and MWCNT networks synergistically enhance the material system’s electrical conductivity. Furthermore, the polymer foam substrates possess high porosity to provide ultra-low thermal conductivity without compromising the electrical conductivity of the TE nanocomposites. With an extremely low GnP loading (i.e., ~1.5 vol.%), the macroporous PVDF nanocomposites exhibit a thermoelectric figure-of-merit of ~10^−3^. To the best of our knowledge, this *ZT* value is the highest value reported for organic TE materials using non-conducting polymers and MWCNT/GnP nanofillers. The proposed technique represents an industrially viable approach to fabricate organic TE materials with enhanced energy conversion efficiencies. The current study demonstrates the potential to develop light-weight, low-cost, and flexible TE materials for green energy generation.

## 1. Introduction

During recent decades, thermoelectric (TE) materials have attracted significant research interest for renewable and green energy applications [1,2]. These materials can directly convert thermal energy into electrical energy, owing to the Seebeck effects. By imposing a small temperature difference across a TE material, charge carriers (i.e., electrons or holes) will diffuse between the hot and cold sides and create an electric current. This phenomenon can be utilized to harvest energy from waste heat given by different sources and to improve the efficiency of many power generating systems [3,4]. Moreover, thermoelectric generators (TEGs) have many advantages over conventional power generators (i.e., combustion engines and turbines) such as lack of pollution, silent operation, and reliability. The energy conversion efficiencies of TE materials are usually measured by a dimensionless figure of merit (*ZT*),

*ZT* = *σS*^2^*T*/*k* (1)

where *σ* is the electrical conductivity (S cm^−1^), *S* is the Seebeck coefficient (µV K^−1^), *k* is the thermal conductivity (W m^−1^K^−1^), and *T* is the absolute temperature (K). Therefore, a high *ZT* value can be achieved by increasing the power factor (*PF*), which is expressed as *σS^2^*, while decreasing the thermal conductivity of the material. For electrically conductive materials the *k* comprises of two parts as expressed in Equation (2).

*k* = *k_l_* + *k_e_* (2)

where *k_l_* is the lattice thermal conductivity due to phonon transferring and *k_e_* is the electronic thermal conductivity due to electron movements [5]. According to the Wiedemann-Franz law, *k_e_* is correlated with the electrical conductivity of the material through Equation (3).

*k_e_* = *σLT* (3)

where *L* is the Lorenz number [6]. Consequently, decreasing the lattice thermal conductivity is the best way to suppress *k* without compromising *σ* in order to maximize the *ZT* value. The electrical conductivities of materials, on the other hand, usually have an inverse relationship with their Seebeck coefficients [7]. Therefore, a fundamental challenge to improve a material’s TE efficiency is the need to decouple the interrelated TE parameters (i.e., *σ*, *S*, and *k*) [8]. Since the discovery of the thermoelectric effect, various processing techniques have been developed to enhance the TE efficiency of different classes of materials [9,10]. Recent advances in nanotechnology have opened a new route to partially decouple the TE properties of nanostructured materials [11,12]. Introducing nano-interfaces enhances phonon scattering within the bulk of materials without significantly affecting their electron transfer. Fabricating small dimensional material structures such as quantum wells, quantum dots, and thin films has been demonstrated as an effective strategy to enhance the Seebeck coefficient of TE materials. The quantum confinement effects of nanostructured materials help to alter their electronic density of states, leading to enhanced Seebeck coefficients [13,14,15]. 

Semiconductors (e.g., bismuth telluride alloys) are commonly used for TE applications due to their high Seebeck coefficients and relatively good electrical conductivities that result in their high *ZT* values (i.e., ~1 to 2). However, because of their toxicity, scarcity, and high costs, semiconducting TE materials have found limited industrial applications. Organic TE materials have recently drawn more attention as potential alternatives to semiconductors for TE applications due to their many advantages such as flexibility, light-weight, low cost, good processability, and environmental sustainability [16,17]. Their intrinsic low thermal conductivities are also desirable to achieve high *ZT* values. In contrast, the electrical conductivity and Seebeck coefficient of polymers should be significantly improved to make them viable options for TE applications. Conducting fillers, such as metallic or carbon particles, have been incorporated within polymeric matrices to enhance their electrical conductivity [18,19,20]. However, the addition of conducting fillers would also compromise the material system’s low thermal conductivity, which would negatively affect their TE efficiencies [21,22].

Conjugated polymers or intrinsically conducting polymers (ICPs) are among the most investigated organic materials for TE applications. The electrical conductivity of conjugated polymers can reach the levels of semiconducting materials through different doping mechanisms [23]. However, because of their low stability, especially at high temperatures, conducting polymers have found limited TE applications [24]. Recent studies suggested graphene and carbon nanotube as promising candidates to improve the TE properties of polymer material systems [25,26]. By controlling the dispersion of carbon nanofillers within polymer matrices, continuous 3D networks can be created to facilitate electron transfer and promote the electrical conductivity of polymer nanocomposites [27,28]. Moreover, creating filler-filler junctions and organic-inorganic interfaces in polymer nanocomposites has demonstrated great potential to increase their Seebeck coefficients through a carrier filtering effect, while suppressing their thermal conductivity via phonon scattering [29,30,31]. Hybridization of metallic, carbon-based, and semiconducting fillers has also proven to be capable of enhancing the *ZT* values of polymer nanocomposites by simultaneously tuning their TE parameters [32,33,34]. Our previous study showed that incorporating closed-cell structures within polymer nanocomposites could alter the localization of nanofillers and thereby controlled the formation of electrically conductive pathways in the polymer matrices [35]. Thermally insulating air voids in nanocomposite foams also significantly suppressed their thermal conductivities. This led to further improvement in their TE efficiencies.

In this study, a new processing technique is proposed to fabricate TE polymer nanocomposites with enhanced energy conversion efficiencies. This approach uses macroporous polymer foam templates with open cellular structures to assist the formation of conductive filler networks within polymer matrices. Layer-by-layer (LBL) deposition of multi-walled carbon nanotubes (MWCNTs) and/or graphene nanoplatelets (GnPs) throughout the cellular structures of polyvinylidene fluoride (PVDF) foams would result in segregated networks of conducting fillers to transfer charge carriers throughout the material system. The effects of using 1D and 2D conducting nanofillers, filler hybridization, and filler contents on TE properties (i.e., electrical conductivity, thermal conductivity, and Seebeck coefficient) of nanocomposite foams have been thoroughly investigated to optimize their TE efficiencies.

## 2. Materials and Methods

### 2.1. Fabrication of Macroporous PVDF Templates

Polyvinylidene fluoride (PVDF, Kynar 740, Arkema, Colombes, France) was used to fabricate polymeric foam templates via a salt-leaching method. A typical thermoplastic polymer was selected in this study due to its ease of processing, good mechanical strength, and flexibility to serve as a template for fabricating TE polymer nanocomposite samples. PVDF powders were dry-blended with sodium chloride (NaCl) salt with particle sizes ranging from 250 to 500 µm. PVDF-NaCl samples were made by hot-pressing the mixture at 185 °C and 4000 psi into disc-shaped molds of 20 mm in diameter and 2 mm in thickness. PVDF-NaCl composite samples were immersed in a water bath at room temperature (i.e., 23 °C) for 72 h to leach out the salt content and produce macroporous PVDF foam templates with open-cellular structures. Fabricated PVDF foams were dried in an oven at 70 °C for 12 h and weighed to ensure complete removal of their salt contents. A high percentage of salt particles within the PVDF-NaCl mixtures was crucial in this fabrication technique. It allowed the complete removal of all NaCl and achieved open cellular foam structures. The effects of different foam structures on the thermal conductivities and filler adsorption abilities of PVDF templates were investigated in our previous work [36]. In this study, the PVDF foam templates were fabricated using 90 wt.% of NaCl to achieve high porosity with excessive specific surface areas. This made them ideal templates for absorbing carbon nanoparticles to form continuous filler networks.

### 2.2. Multilayer Deposition of GnP-MWCNT Network

Nanocomposite samples were prepared by layer-by-layer (LBL) deposition of conducting nanofillers on the cell-walls of as-fabricated polymeric foam templates prepared by the salt leaching method. Multi-walled carbon nanotubes (MWCNT, 1.0 wt.% aqueous dispersed, AQ0101, Nanocyl, Sambreville, Belgium) and graphene nanoplatelets (GnP, Grade 2, CheapTubes, Cambridgeport, MA, USA) were used as conducting nanoparticles to fabricate polymer nanocomposite samples. As-fabricated PVDF foams were repeatedly immersed into aqueous solutions of carbon nanofillers followed by drying in an oven (VWR^®^ Vacuum Oven, VWR^®^, Pennysylvania, PA, USA) at 70 °C for 12 h to coat multiple layers of nanoparticles over the PVDF porous structures. Each coating cycle was performed by sonicating the templates in the filler solutions for two minutes using an ultrasonic probe (Q700, QSonica, Newtown, CT, USA). This would facilitate the penetration of nanoparticles throughout the pores in PVDF templates and create a uniform coating of nanoparticles on their cell walls. 

An aqueous solution of GnPs was prepared using sodium dodecyl sulfate (SDS, Sigma-Aldrich, St. Louis, MO, USA) as the surfactant, with a GnP:SDS mass ratio of 1, to ensure a stable dispersion of GnP. Solutions of MWCNT-GnP mixtures with MWCNT:GnP mass ratios of 1 and 0.1 were also prepared to fabricate nanocomposite samples filled with hybrid GnP-MWCNT fillers. The solutions were sonicated for 10 min to ensure uniform dispersion of nanoparticles. The total filler content of all the solutions was gradually increased after each coating cycle from 0.1 wt.% to 1 wt.% in order to achieve the desired coating level and ensure proper attachment of fillers onto the cell walls. The adsorption of MWCNTs and/or GnPs onto the interior surfaces of the interconnected pores within PVDF foams would yield a multilayered and continuous network of conductive nanoparticles throughout the polymer templates. The experimental results revealed that fewer coating cycles would be needed to achieve the desired filler loading level while using MWCNTs compared with GnPs or hybrid fillers. This is because of the 1D and wavy structure of MWCNTs which provides better entanglement during their multilayered stacking within polymeric foam templates.

### 2.3. Sample Characterization

Scanning electron microscopy (SEM: Quanta 3D FEG, FEI Company, Hillsboro, AL, USA) was used to analyze the surface morphologies and microstructures of nanocomposite foams. The cross-sections of nanocomposite foams were exposed by cryo-fracturing the samples under liquid nitrogen. The fractured surfaces were sputter-coated by gold using a sputter-coating machine (Desk V Sputter Coater, Denton Vacuum, Moorestown, NJ, USA). The electrical conductivities of nanocomposite foams were measured by the four-point method using a multifunctional source meter (SMU 2450, Keithley, Cleveland, UT, USA) and a collinear four-point probe (SP4 probe head, Signatone, Gilroy, CA, USA) installed on a probing fixture (Probe S-302-4, Signatone, Gilroy, CA, USA). A constant electric current was input on the surfaces of all the fabricate nanocomposite samples, while the measured voltage was recorded to calculate their resistances. A very small current level (i.e., 1 mA) was selected to avoid joule heating within the samples, which could lead to measurement errors. Using ASTM F84-02 standard [37], the sample’s surface electrical conductivity was converted into the bulk conductivity value by using the size and thickness correction factors for each sample. The thermal conductivities of the nanocomposite foams were measured based on the modified transient plane source (MTPS) method using a thermal conductivity analyzer (TCi Thermal Conductivity Analyzer, C-Therm Technologies Ltd., Fredericton, NB, Canada,). The Seebeck coefficient was measured using a custom-made unit. By applying a temperature difference (∆*T*) across the sample’s surface within the range of 2 to 4 °C, the generated voltage (*V_TE_*) was recorded by a source meter. The Seebeck coefficient was calculated from the slope of the *V_TE_* versus ∆*T* plot. All the thermoelectric parameters of the fabricated samples were measured at room temperature (i.e., 300 K) to provide consistent and comparable results. 

## 3. Results and Discussion

### 3.1. Open-Cellular Morphologies of Macroporous PVDF Templates

**Figure 1:**
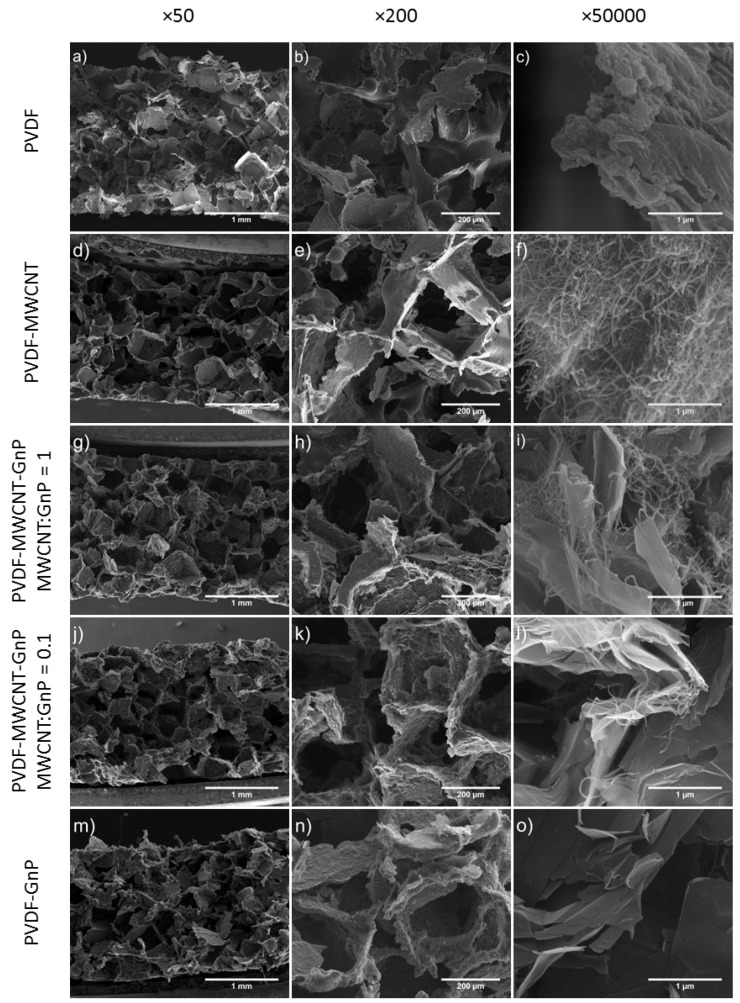
Scanning electron microscopy (SEM) micrographs that illustrate the foam and phase morphologies of: (**a**–**c**) polyvinylidene fluoride (PVDF) foams; (**d**–**f**) PVDF-MWCNT nanocomposites; (**g**–**i**) PVDF-MWCNT-GnP nanocomposites with MWCNT:GnP ratio of 1; (**j**–**l**) PVDF-MWCNT-GnP nanocomposites with MWCNT:GnP ratio of 0.1; and (**m**–**o**) PVDF-GnP nanocomposites at three different magnifications. Note: multi-walled carbon nanotube (MWCNT) and graphene nanoplatelets (GnP).

Figure 1 illustrates the SEM micrographs of PVDF foams, before and after coating with carbon nanofillers, at different magnifications. The open-cellular foam morphologies of all samples revealed the high levels of porosity and interconnectivity of pores throughout the polymeric templates. The SEM micrographs also indicated that after depositing a multilayered structure of carbon nanofillers, the open-cellular structures remained nearly intact and the open-cellular structures of the foam templates were retained.

### 3.2. Phase Morphology of Macroporous PVDF Templates and Their Nanocomposites

The phase morphologies of the nanocomposite foams are demonstrated in SEM micrographs with higher magnifications in Figure 1. The micrographs revealed a uniform coating of conductive fillers on the interior pore surfaces of PVDF foams while carbon nanoparticles were interconnected and evenly adhered to the cell walls. This was achieved as filler solutions were able to penetrate throughout the entire cellular structures of the templates due to their open-cellular structures and high porosities. Figure 1f illustrates that the PVDF-MWCNT nanocomposite sample had a continuous fibrous layer of MWCNT thoroughly covering the cell walls of the PVDF foam. As shown in Figure 1o, the PVDF-GnP nanocomposite sample demonstrated a different surface morphology caused by the stacked layers of GnPs deposited on top of the PVDF template. 

Figure 1i,l show the interactions between MWCNTs and GnPs within the PVDF nanocomposites containing hybrid fillers with MWCNT:GnP ratios of 1 and 0.1, respectively. In PVDF-MWCNT-GnP nanocomposite samples with a MWCNT:GnP ratio of 1, MWCNTs were widespread all over the samples because of their high aspect ratios compared with GnPs. Consequently, MWCNTs were covering most of the surface areas of GnPs while MWCNT agglomerations were observed on templates’ surfaces. On the other hand, in PVDF-MWCNT-GnP nanocomposites with a MWCNT:GnP ratio of 0.1, MWCNTs were scattered on GnPs’ surfaces while bridging along the surfaces and the gaps of adjacent graphene nanoplatelets. Therefore, hybridization of 1D and 2D nanofillers with a low 1D:2D filler ratio created more connected pathways for electron transfer throughout the polymer matrix.

### 3.3. Electrical Conductivity of Macroporous PVDF Nanocomposites

**Figure 2:**
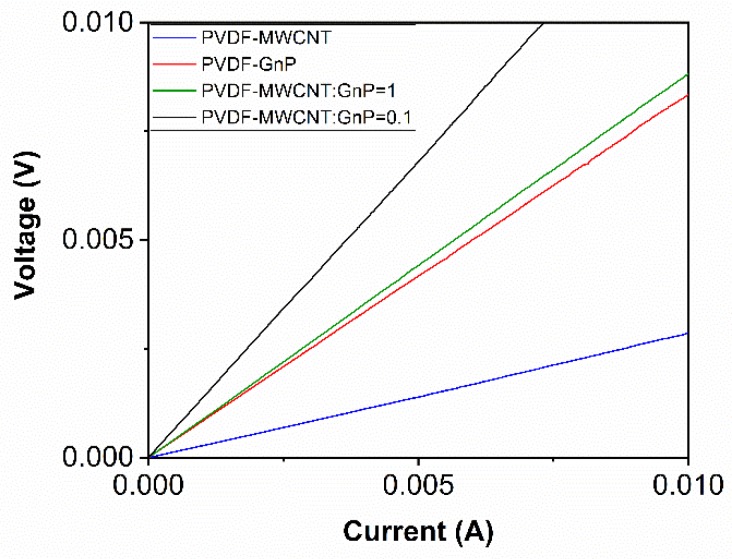
The voltage-current relationship of PVDF nanocomposite samples loaded with about 1.4 wt.% of various carbon nanofillers.

The voltage-current graph for the PVDF nanocomposite samples containing different nanofillers is plotted in Figure 2. As a case example, the samples loaded with about 1.4 wt.% of carbon fillers were selected to investigate their conductivity by measuring the voltage as the current varied. All samples showed linear voltage-current relationships, which represented their ohmic behaviors within the applied current range. The electrical conductivities of the samples could be determined from the slope of their *V-I* graphs.

**Figure 3:**
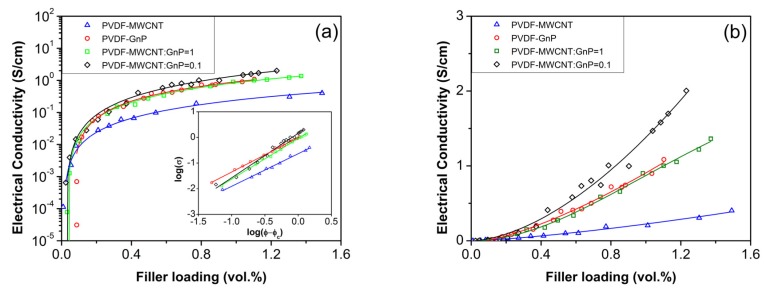
The electrical conductivities of PVDF nanocomposite samples as a function of filler loadings: (**a**) a logarithmic plot showing the percolation behaviors of *σ* with increasing filler loadings; and (**b**) a linear plot demonstrating differences in *σ* values of PVDF nanocomposites.

Figure 3a plots the electrical conductivity of polymer nanocomposites, containing different carbon nanoparticles, as a function of volume percent of nanofillers within PVDF templates. The weight percent (wt.%) of nanofillers loaded on PVDF foams was obtained by measuring the weight of samples before and after each coating cycle. The volume fraction (vol.%) of carbon fillers within PVDF templates were calculated using their wt.% and by taking into account the total volume of voids and the solid part of foams. The densities of MWCNTs and GnPs were considered to be 1.75 g cm^−3^ (for NC7000 type of MWCNTs) and 2.2 g cm^−3^ (a typical reported value for GnPs in literature), respectively [38,39]. By increasing the filler loadings of nanocomposite samples, their electrical conductivities showed an initial sharp increase followed by a gradual improvement at higher filler loadings. This behavior indicated that a percolated network of conducting nanoparticles had been established. The percolation behavior for the electrical conductivity typically follows a power law relationship as expressed in Equation (4).

*σ* ∝ (*ϕ* − *ϕ_c_*)*^t^* (4)

where *σ* is the electrical conductivity, *ϕ* is the filler content, *ϕ_c_* is the percolation threshold, and *t* is a critical exponent reflecting the dimensionality of the composite system [40]. The log(*σ*) versus log(*ϕ − ϕ_c_*) plot for the experimental measurements of all nanocomposite samples, as shown in the inset of Figure 3a, showed a linear trend representing the percolation behaviors. According to Table 1, the nanocomposite foams containing only MWCNTs had the lowest percolation threshold, which was consistent with the results reported in literature [41,42]. The higher aspect ratios of MWCNTs increased their chances of creating conducting networks within the polymeric matrix at low filler contents. The results also revealed that the calculated percolation thresholds of polymeric foams containing hybrid nanofillers were at least 50% lower than PVDF-GnP samples. At low filler loadings, MWCNTs helped to bridge among scattered GnP fillers, and thereby facilitated the creation of percolated pathways for electron transfer throughout the PVDF foam templates.

As illustrated in Figure 3a, increasing the nanofiller loadings above 0.1 vol.% significantly enhanced the electrical conductivity of nanocomposite foams containing GnPs and surpassed that of PVDF-MWCNT samples. Figure 3b plots the electrical conductivity of the fabricated samples on a linear scale to clearly demonstrate the difference in *σ* values of different nanocomposite samples. With 1.0 vol.% filler content, the electrical conductivity of PVDF-GnP samples was 3.5 times higher than that of the PVDF-MWCNT nanocomposites. This indicated that GnPs provided more efficient electron transferring pathways within the polymer matrix. Similar results were reported in literature comparing the electrical conductivity of polymer nanocomposites prepared with GnPs, MWCNTs, or a mixture of them [43,44]. This could be attributed to their 2D structures, which provided high electron mobility and strong filler interactions [45]. It should be noted that the overall electrical conductivity of the nanocomposite samples was much lower than the measured values for pure MWCNT and GnP samples (i.e., 30 S/cm for a MWCNT film prepared by solution casting, and 74 S/cm for a GnP solid sample prepared by cold pressing) due to the electrical contact resistance at the introduced filler intersections.

Experimental results also revealed that hybrid nanocomposite foams with a MWCNT:GnP ratio of 0.1 showed the highest electrical conductivity values among all the nanocomposite samples. The omnipresence of MWCNTs among GnPs helped to bridge adjacent platelets and thereby promoted the formation of interconnected networks throughout the insulating PVDF matrix. Therefore, simultaneously utilizing the high intrinsic electrical conductivity of GnPs and the low percolation threshold of MWCNTs promoted the nanocomposite’s electrical conductivity. However, with a MWCNT:GnP ratio of 1, MWCNTs were present all over PVDF’s cell walls and created more agglomerated domains on GnPs’ surfaces. As a result, MWCNT fillers with their inferior electrical conductivities adversely affected the electron transfer among GnPs.

### 3.4. Thermal Conductivities of Macroporous PVDF Nanocomposites

**Figure 4:**
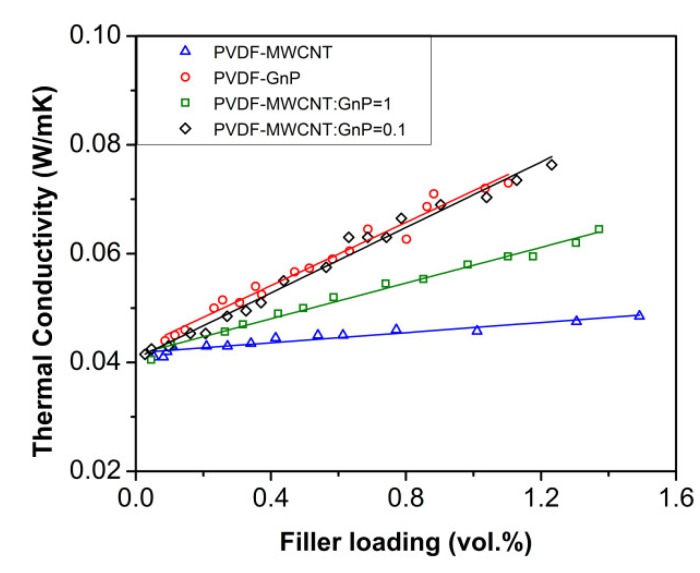
Thermal conductivities of PVDF nanocomposites as a function of filler loadings.

The effect of filler contents on the thermal conductivities (*k*) of all nanocomposite foams is depicted in Figure 4. As expected, the thermal conductivity of open-cellular PVDF templates without nanoparticle coating was extremely low (i.e., ~0.04 W m^−1^K^−1^) because of their high porosity.

Experimental results indicated that the deposition of MWCNTs on PVDF templates had negligible effects on their thermal conductivity, while PVDF foams containing GnPs showed a slight increase in their *k*. GnPs have reportedly better thermal transport properties compared with MWCNTs because of their unique 2D atomic structures [46,47,48]. Nevertheless, the overall thermal conductivities of all PVDF nanocomposites remained very low (i.e., *k* < 0.08 W m^−1^K^−1^) despite the adsorption of up to 15 wt.% nanoparticles. The introduced interfacial thermal resistance at filler-filler junctions due to phonon scattering along with the high porosity of the PVDF templates helped to retain extra-low thermal conductivities of the nanocomposite foams [49]. Consequently, the measured thermal conductivities of PVDF nanocomposites were significantly lower than the typical values for polymer nanocomposites containing carbon fillers (i.e., ~0.5–1 W m^−1^K^−1^) [50].

### 3.5. Seebeck Coefficient of Macroporous PVDF Nanocomposites

**Figure 5:**
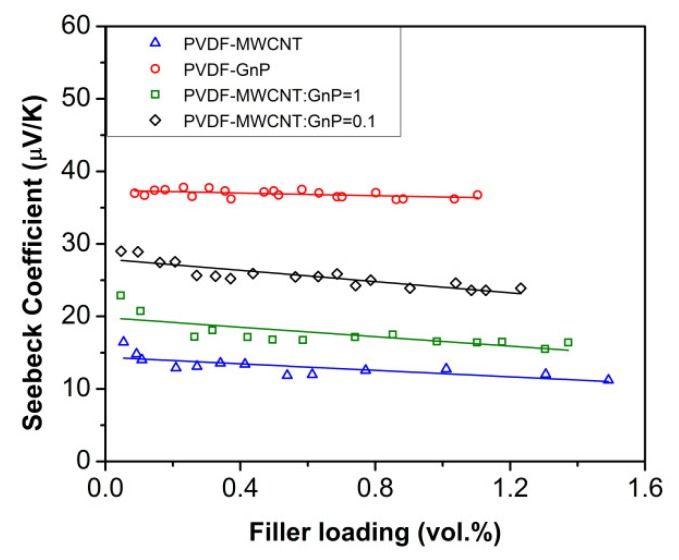
Seebeck coefficients of PVDF nanocomposites foams as a function of filler loadings.

The effects of filler-type and filler content on the Seebeck coefficients (*S*) of the fabricated nanocomposite foams are illustrated in Figure 5. All nanocomposite samples had positive Seebeck values indicating a p-type thermoelectric behavior. MWCNT and GnP fillers have reportedly shown p-type thermoelectric behavior because of their oxygen-doping during exposure to the air. The experiments revealed that the Seebeck coefficients of the nanocomposite foams were significantly higher than the measured values for pure conducting fillers (i.e., *S* < 8 µV K^−1^ for MWCNT film prepared by solution casting, and *S* < 11 µV K^−1^ for GnP solid sample prepared by cold pressing). This phenomenon could be attributed to the energy filtering effect aroused from charge carrier scattering at the significantly large number of filler junctions throughout the pore surfaces of the open-cellular templates [51,52].

The highest Seebeck coefficients of PVDF nanocomposites were achieved once their filler loadings had reached their percolation thresholds. By increasing the filler loadings of nanocomposite foams, their Seebeck coefficients initially dropped and thereafter remained around the same range. The reduction in their Seebeck coefficients was expected as higher filler loadings led to enhanced electrical conductivity and there is usually a trade-off between *σ* and *S* values in TE materials. It should be noted that the decrease in the Seebeck coefficients of all nanocomposite foams was negligible compared to the increase in their electrical conductivities (i.e., Δ*S* < 30% vs. Δ*σ* > 10^3^ times). The increased interfacial interaction between conducting nanofillers, as a result of the layer-by-layer coating process, helped to retain high Seebeck coefficients of nanocomposite samples while increasing their electrical conductivities. The PVDF-GnP foams demonstrated the highest Seebeck values among all the nanocomposite samples (i.e., ~37 µV K^−1^) and their Seebeck coefficients were three times higher than those of PVDF-MWCNT samples. The experimental results for the nanocomposite samples loaded with hybrid fillers also revealed that a lower MWCNT:GnP ratio was more favorable to achieve high Seebeck coefficients. These results indicated that GnPs have better Seebeck properties than MWCNTs.

### 3.6. TE Figure-of-Merit of Macroporous PVDF Nanocomposites

**Figure 6:**
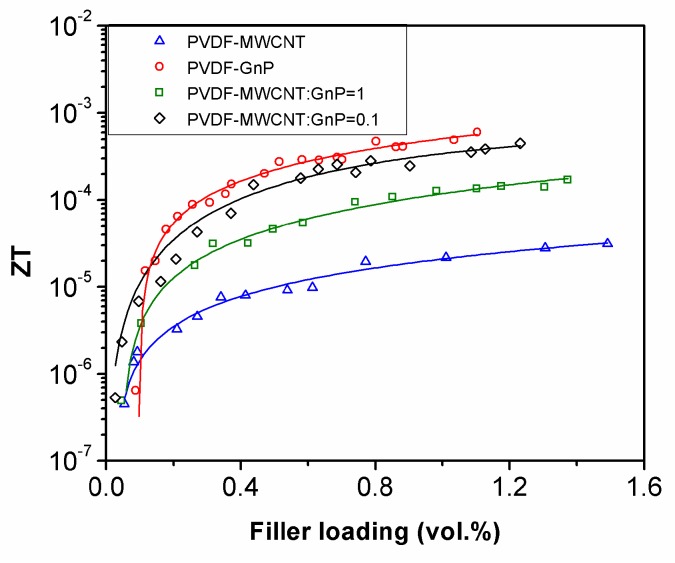
Thermoelectric (TE) efficiencies of PVDF nanocomposite foams as a function of filler loadings.

The measured *ZT* values of the fabricated nanocomposite samples are presented in Figure 6. By increasing the filler contents of the polymeric foams, their TE efficiencies significantly improved showing a percolation behavior. At high filler loadings, PVDF-GnP nanocomposite samples demonstrated the highest *ZT* values among all fabricated samples due to their high Seebeck coefficients. Despite the highest measured electrical conductivities, the *ZT* values of PVDF foams containing hybrid nanofillers with a MWCNT:GnP ratio of 0.1 were slightly lower than those of PVDF-GnP samples because of their lower Seebeck coefficients. At low filler loadings (i.e., <0.1 vol.%), however, PVDF-GnP nanocomposites showed the lowest TE efficiencies due to their low electrical conductivities. Among the three TE parameters (i.e., *σ*, *S*, and *k*) of the fabricated samples, the changes in their electrical conductivities during nanoparticle deposition were more noticeable. Therefore, was the decisive parameter that defined the *ZT* values of PVDF nanocomposites.

**Figure 7:**
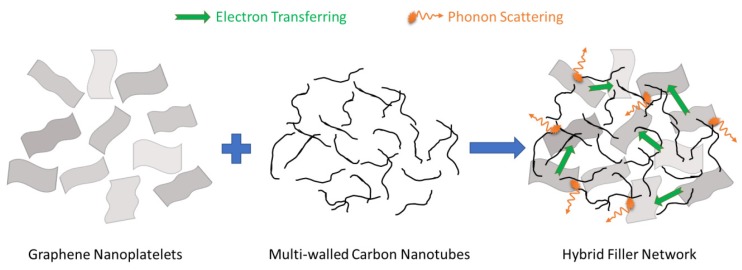
The synergistic effects of 1D and 2D conducting nanoparticles on electron and phonon transferring through their 3D networks.

Experimental results suggested that GnP was the more promising candidate than MWCNT for fabricating TE polymer nanocomposites. The observations indicated that the TE efficiencies of the nanocomposite samples containing hybrid fillers could potentially be maximized by optimizing the MWCNT:GnP mass ratio and utilizing the interactions between 1D and 2D conducting nanoparticles. Figure 7 shows a schematic diagram, illustrating the synergistic effects of the hybridization of 1D and 2D nanoparticles. Loosely packed hybrid MWCNT-GnP fillers can facilitate electron transfer through their 3D networks without compromising the Seebeck coefficients due to the carrier filtering effect at the filler-filler interfaces. Moreover, this 3D filler network can suppress the thermal conductivity through phonon scattering at multiple filler junctions.

This study proposed the utilization of polymer foam templates as a new fabrication strategy to partially decouple TE properties of polymer nanocomposite materials and promote their TE efficiencies. A maximum *ZT* value approaching 10^−3^ was achieved by PVDF nanocomposites containing extremely low loadings (i.e., <1.5 vol.%) of carbon nanofillers. This value is among the highest of the *ZT* values reported in literature for organic TE materials using conventional non-conducting polymers. It is believed that by using the suggested fabrication technique and utilizing conducting fillers with better *σ* and *S* properties (e.g., single-walled carbon nanotubes (SWCNTs) or semiconducting fillers), further increase in *ZT* values would undoubtedly be achieved for polymeric TE materials.

## 4. Conclusions

A new technique has been developed to fabricate lightweight organic materials with high TE efficiencies. Polymeric foams with open-cellular structures were employed as templates to create segregated networks of conducting nanofillers within the insulating polymer matrices. The resultant polymer nanocomposite foams demonstrated low percolation thresholds and high electrical conductivities, without compromising the ultra-low thermal conductivities of the PVDF foam templates. The synergistic effect of 1D and 2D carbon nanoparticles in promoting the TE properties of polymer nanocomposites containing hybrid fillers was also investigated in this study. Experimental results revealed that by controlling the MWCNT:GnP mass ratio incorporated in polymeric matrices, it was possible to tune their electrical conductivities, Seebeck coefficients, and percolation thresholds. The proposed processing method has proven to be a facile and effective approach to simultaneously improve the electrical conductivities, enhance the Seebeck coefficients, and suppress the thermal conductivities of polymer nanocomposites. Overall, it helps to promote the TE efficiency of polymer nanocomposites without the uses of conducting polymers that have low processability. The suggested fabrication method is facile and scalable for processing flexible TE nanocomposites, which increases the potential of their industrial applications.

## Figures and Tables

**Table 1:** The percolation thresholds and the critical exponents of PVDF nanocomposite foams coated with different types of carbon nanoparticles.

Sample	PVDF-MWCNT	PVDF-MWCNT-GnP MWCNT:GnP = 1	PVDF-MWCNT-GnP MWCNT:GnP = 0.1	PVDF-GnP
Percolation Threshold (*ϕ_c_*)	0.009	0.032	0.024	0.065
Critical Exponent (*t*)	1.40	1.45	1.63	1.36

## References

[1] Hamid Elsheikh M., Shnawah D.A., Sabri M.F.M., Said S.B.M., Haji Hassan M., Ali Bashir M.B., Mohamad M. (2014). A Review on thermoelectric renewable energy: Principle parameters that affect their performance. Renew. Sustain. Energy Rev..

[2] Zheng X.F., Liu C.X., Yan Y.Y., Wang Q. (2014). A review of thermoelectrics research–recent developments and potentials for sustainable and renewable energy applications. Renew. Sustain. Energy Rev..

[3] Bell L.E. (2008). Cooling, heating, generating power, and recovering waste heat with thermoelectric systems. Science.

[4] Riffat S., Ma X. (2003). Thermoelectrics: A review of present and potential applications. Appl. Therm. Eng..

[5] Wan C., Wang Y., Wang N., Norimatsu W., Kusunoki M., Koumoto K. (2010). Development of novel thermoelectric materials by reduction of lattice thermal conductivity. Sci. Technol. Adv. Mater..

[6] Snyder G.J., Toberer E.S. (2008). Complex thermoelectric materials. Nat. Mater..

[7] Minnich A.J., Dresselhaus M.S., Ren Z.F., Chen G. (2009). Bulk nanostructured thermoelectric materials: Current research and future prospects. Energy Environ. Sci..

[8] Xiao C., Li Z., Li K., Huang P., Xie Y. (2014). Decoupling interrelated parameters for designing high performance thermoelectric materials. Acc. Chem. Res..

[9] Alam H., Ramakrishna S. (2013). A Review on the enhancement of figure of merit from bulk to nano-thermoelectric materials. Nano Energy.

[10] Chen G., Dresselhaus M.S., Dresselhaus G., Fleurial J.P., Caillat T. (2003). Recent developments in thermoelectric materials. Int. Mater. Rev..

[11] Martín-González M., Caballero-Calero O., Díaz-Chao P. (2013). Nanoengineering thermoelectrics for 21st century: Energy harvesting and other trends in the field. Renew. Sustain. Energy Rev..

[12] Chen Z.G., Han G., Yang L., Cheng L., Zou J. (2012). Nanostructured thermoelectric materials: current research and future challenge. Prog. Nat. Sci. Mater. Int..

[13] Pei Y., Wang H., Snyder G.J. (2012). Band engineering of thermoelectric materials. Adv. Mater..

[14] Mao J., Liu Z., Ren Z. (2016). Size Effect in thermoelectric materials. npj Quantum Mater..

[15] Venkatasubramanian R., Siivola E., Colpitts T., O’Quinn B. (2001). Thin-film thermoelectric devices with high room-temperature figures of merit. Nature.

[16] Zhang Q., Sun Y., Xu W., Zhu D. (2014). Organic Thermoelectric materials: emerging green energy materials converting heat to electricity directly and efficiently. Adv. Mater..

[17] Culebras M., Gómez C., Cantarero A. (2014). Review on polymers for thermoelectric applications. Materials.

[18] Gangopadhyay R., De A. (2000). Conducting polymer nanocomposites: A brief overview. Chem. Mater..

[19] Piao M., Kim G., Kennedy G.P., Roth S., Dettlaff-Weglikowska U. (2013). Preparation and characterization of expanded graphite polymer composite films for thermoelectric applications. Phys. Status Solidi.

[20] Luo J., Krause B., Pötschke P. (2016). Melt-mixed thermoplastic composites containing carbon nanotubes for thermoelectric applications. AIMS Mater. Sci..

[21] Winey K.I., Kashiwagi T., Mu M. (2007). Improving electrical conductivity and thermal properties of polymers by the addition of carbon nanotubes as fillers. MRS Bull..

[22] Veca L.M., Meziani M.J., Wang W., Wang X., Lu F., Zhang P., Lin Y., Fee R., Connell J.W., Sun Y.P. (2009). Carbon nanosheets for polymeric nanocomposites with high thermal conductivity. Adv. Mater..

[23] Dubey N., Leclerc M. (2011). Conducting polymers: efficient thermoelectric materials. J. Polym. Sci. Part B Polym. Phys..

[24] Taroni P.J., Hoces I., Stingelin N., Heeney M., Bilotti E. (2014). Thermoelectric materials: A brief historical survey from metal junctions and inorganic semiconductors to organic polymers. Isr. J. Chem..

[25] Dey A., Bajpai O.P., Sikder A.K., Chattopadhyay S., Shafeeuulla Khan M.A. (2016). Recent advances in cnt/graphene based thermoelectric polymer nanocomposite: A proficient move towards waste energy harvesting. Renew. Sustain. Energy Rev..

[26] Gao C., Chen G. (2016). Conducting polymer/carbon particle thermoelectric composites: Emerging green energy materials. Compos. Sci. Technol..

[27] Yu C., Kim Y.S., Kim D., Grunlan J.C. (2008). Thermoelectric behavior of segregated-network polymer nanocomposites. Nano Lett..

[28] Gorrasi G., Bugatti V., Milone C., Mastronardo E., Piperopoulos E., Iemmo L., Di Bartolomeo A. (2018). Effect of temperature and morphology on the electrical properties of PET/conductive nanofillers composites. Compos. Part B Eng..

[29] He M., Ge J., Lin Z., Feng X., Wang X., Lu H., Yang Y., Qiu F. (2012). Thermopower enhancement in conducting polymer nanocomposites via carrier energy scattering at the organic–inorganic semiconductor interface. Energy Environ. Sci..

[30] Wang Y., Liu J., Zhou J., Yang R. (2013). Thermoelectric transport across nanoscale polymer–semiconductor–polymer junctions. J. Phys. Chem. C.

[31] Zhang K., Zhang Y., Wang S. (2013). Enhancing thermoelectric properties of organic composites through hierarchical nanostructures. Sci. Rep..

[32] Yang K., Chen Y., D’Agosta R., Xie Y., Zhong J., Rubio A. (2012). Enhanced thermoelectric properties in hybrid graphene/boron nitride nanoribbons. Phys. Rev. B.

[33] Pang H., Piao Y.Y., Tan Y.Q., Jiang G.Y., Wang J.H., Li Z.M. (2013). Thermoelectric behaviour of segregated conductive polymer composites with hybrid fillers of carbon nanotube and bismuth telluride. Mater. Lett..

[34] Zhang K., Wang S., Zhang X., Zhang Y., Cui Y., Qiu J. (2015). Thermoelectric performance of p-type nanohybrids filled polymer composites. Nano Energy.

[35] Aghelinejad M., Leung S.N. (2017). Enhancement of thermoelectric conversion efficiency of polymer/carbon nanotube nanocomposites through foaming-induced microstructuring. J. Appl. Polym. Sci..

[36] Aghelinejad M., Leung S.N. (2018). Fabrication of open-cell thermoelectric polymer nanocomposites by template-assisted multi-walled carbon nanotubes coating. Compos. Part B Eng..

[37] ASTM F84-02 (2002). Standard Test Method for Measuring Resistivity of Silicon Wafers With an In-Line Four-Point Probe (Withdrawn 2003).

[38] Ameli A., Nofar M., Park C.B., Pötschke P., Rizvi G. (2014). Polypropylene/Carbon nanotube nano/microcellular structures with high dielectric permittivity, low dielectric loss, and low percolation threshold. Carbon.

[39] Ma P.C., Siddiqui N.A., Marom G., Kim J.K. (2010). Dispersion and functionalization of carbon nanotubes for polymer-based nanocomposites: A review. Compos. Part A Appl. Sci. Manuf..

[40] Kirkpatrick S. (1973). Percolation and conduction. Rev. Mod. Phys..

[41] Punetha V.D., Rana S., Yoo H.J., Chaurasia A., McLeskey J.T., Ramasamy M.S., Sahoo N.G., Cho J.W. (2017). Functionalization of carbon nanomaterials for advanced polymer nanocomposites: a comparison study between cnt and graphene. Prog. Polym. Sci..

[42] Sun X., Sun H., Li H., Peng H. (2013). Developing polymer composite materials: Carbon nanotubes or graphene?. Adv. Mater..

[43] Kong K.T.S., Mariatti M., Rashid A.A., Busfield J.J.C. (2014). Enhanced conductivity behavior of polydimethylsiloxane (pdms) hybrid composites containing exfoliated graphite nanoplatelets and carbon nanotubes. Compos. Part B Eng..

[44] Kumar S., Sun L.L., Caceres S., Li B., Wood W., Perugini A., Maguire R.G., Zhong W.H. (2010). dynamic synergy of graphitic nanoplatelets and multi-walled carbon nanotubes in polyetherimide nanocomposites. Nanotechnology.

[45] Yoo D., Kim J., Lee S.H., Cho W., Choi H.H., Kim F.S., Kim J.H. (2015). Effects of one- and two-dimensional carbon hybridization of PEDOT:PSS on the power factor of polymer thermoelectric energy conversion devices. J. Mater. Chem. A.

[46] Pop E., Varshney V., Roy A.K. (2012). Thermal properties of graphene: fundamentals and applications. MRS Bull..

[47] Nika D.L., Pokatilov E.P., Askerov A.S., Balandin A.A. (2009). Phonon thermal conduction in graphene: Role of umklapp and edge roughness scattering. Phys. Rev. B.

[48] Kuilla T., Bhadra S., Yao D., Kim N.H., Bose S., Lee J.H. (2010). Recent advances in graphene based polymer composites. Prog. Polym. Sci..

[49] Xu Z., Buehler M.J. (2009). Nanoengineering heat transfer performance at carbon nanotube interfaces. ACS Nano.

[50] Han Z., Fina A. (2011). Thermal conductivity of carbon nanotubes and their polymer nanocomposites: A review. Prog. Polym. Sci..

[51] He M., Qiu F., Lin Z. (2013). Towards high-performance polymer-based thermoelectric materials. Energy Environ. Sci..

[52] Yang J., Yip H.L., Jen A.K.Y. (2013). Rational design of advanced thermoelectric materials. Adv. Energy Mater..

